# Genetic and pharmacological manipulations of the serotonergic system in early life: neurodevelopmental underpinnings of autism-related behavior

**DOI:** 10.3389/fncel.2013.00072

**Published:** 2013-06-12

**Authors:** Karsten Kinast, Deborah Peeters, Sharon M. Kolk, Dirk Schubert, Judith R. Homberg

**Affiliations:** ^1^Behavioural Neurogenetics, Department of Cognitive Neuroscience, Centre for Neuroscience, Donders Institute for Brain, Cognition, and Behaviour, Radboud University Nijmegen Medical CentreNijmegen, Netherlands; ^2^Department of Molecular Animal Physiology, Centre for Neuroscience, Donders Institute for Brain, Cognition, and Behaviour, Radboud University Nijmegen Medical CentreNijmegen, Netherlands

**Keywords:** serotonin, neurodevelopment, ASD, prenatal, social behavior, connectivity, valproic acid, SSRI

## Abstract

Serotonin, in its function as neurotransmitter, is well-known for its role in depression, autism and other neuropsychiatric disorders, however, less known as a neurodevelopmental factor. The serotonergic system is one of the earliest to develop during embryogenesis and early changes in serotonin levels can have large consequences for the correct development of specific brain areas. The regulation and functioning of serotonin is influenced by genetic risk factors, such as the serotonin transporter polymorphism in humans. This polymorphism is associated with anxiety-related symptoms, changes in social behavior, and cortical gray and white matter changes also seen in patients suffering from autism spectrum disorders (ASD). The human polymorphism can be mimicked by the knockout of the serotonin transporter in rodents, which are as a model system therefore vital to explore the precise neurobiological mechanisms. Moreover, there are pharmacological challenges influencing serotonin in early life, like prenatal/neonatal exposure to selective serotonin reuptake inhibitors (SSRI) in depressed pregnant women. There is accumulating evidence that this dysregulation of serotonin during critical phases of brain development can lead to ASD-related symptoms in children, and reduced social behavior and increased anxiety in rodents. Furthermore, prenatal valproic acid (VPA) exposure, a mood stabilizing drug which is also thought to interfere with serotonin levels, has the potency to induce ASD-like symptoms and to affect the development of the serotonergic system. Here, we review and compare the neurodevelopmental and behavioral consequences of serotonin transporter gene variation, and prenatal SSRI and VPA exposure in the context of ASD.

## Introduction

**Figure 1:**
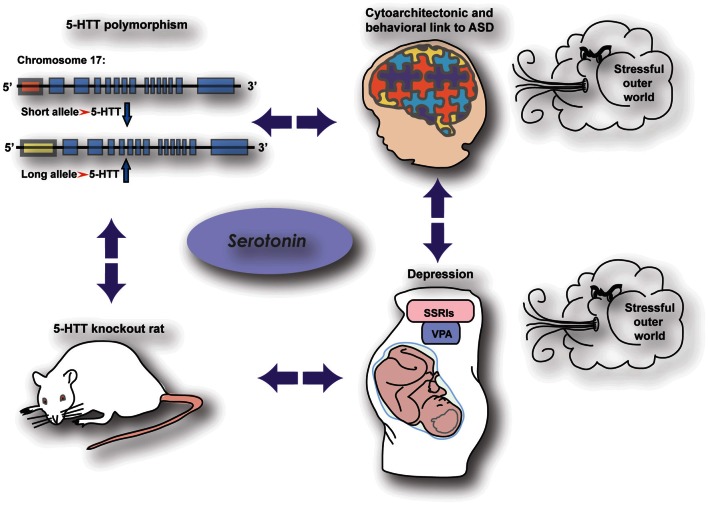
**The relationship between serotonergic genetic and pharmacological manipulations with common effects on brain wiring and behavior**.

Accumulating evidence suggests an important role for the serotonergic system in the onset of mental illnesses in general and autism spectrum disorders in particular (ASD; Box 1). Because of serotonin's (5-HT) ability to modulate developmental processes (Gaspar et al., 2003; Homberg et al., 2010), a modification of the serotonergic system is seen as a crucial factor in the occurrence of dysfunctional developmental programming leading to abnormal behavior in adult life. Therefore, studying the behavioral consequences of early life alterations in the serotonergic system is of major importance to increase our knowledge and understanding of these mental illnesses. There are several possibilities for genetic as well as pharmacological manipulation of the serotonergic system which are of great use to unravel the complex function of 5-HT. Regulation of 5-HT levels can be influenced by genetic factors such as genetic variance in the 5-HT transporter (5-HTT) gene. The most widely studied 5-HTT polymorphism in humans is the 5-HTT Length Polymorphic Region (5-HTTLPR), which involves genetic variance in the promoter region of the 5-HTT gene (Lesch et al., 1996; section The Human 5-HTT Polymorphism) In rodents, this genetic variance is modeled by a mutation of the 5-HTT gene (5-HTT^−/−^) (Kalueff et al., 2010). Although the latter mutation is not promoter specific, the behavioral consequences are very similar compared to those associated with the 5-HTTLPR in humans, including increased anxiety, depression-related behavior in the context of stress, prosocial behavior, and increased behavioral flexibility (Kalueff et al., 2010). There are also pharmacological factors influencing early developmental 5-HT levels, such as selective serotonin reuptake inhibitors (SSRIs). These antidepressant drugs are commonly prescribed to depressed pregnant women and are able to cross the placenta (Homberg et al., 2010; Olivier et al., 2011). SSRIs block the 5-HTT, and thereby give rise to high 5-HT levels not only in the mother but also in the developing fetus. Another agent that may affect 5-HT levels during development is valproic acid (VPA). This drug is used as mood stabilizer and when taken during pregnancy, affects the 5-HT system of the developing brain (Markram et al., 2007). What is particularly interesting is that these genetic and pharmacological factors are all associated with common structural phenotypes in the brain and behavioral manifestations (Figure 1). Hence, comparing the different conditions associated with high 5-HT levels during development (genetic 5-HTT down-regulation, prenatal SSRI and prenatal VPA exposure) may lead to insights relevant for prevention, diagnosis, and/or treatment of ASD, as well as our fundamental understanding of the role of 5-HT in brain development. It is our aim to discuss these three conditions associated with increased 5-HT levels during development in human subjects as well as rodents, and discuss the possible mechanisms underlying the similarities.

Box 1 **Autism Spectrum Disorder (ASD) endophenotypes.**

ASD is a neurodevelopmental disorder manifesting within the first 3 years after birth and progressively worsening in the course of life. Core symptoms of ASD are impairments in sociability (no interest in interaction with others, dysfunction in managing complex social interactions), communicative skills and imagination (absence of spoken language or mild language impairments), and repetitive behavioral patterns (stereotype, preference for sameness, complex rituals (American Psychiatric Association, 1994). Additionally, ASD patients show abnormalities in perception, attention and memory (Ben Shalom, 2003; Dakin and Frith, 2005), as well as increased anxiety (potentially as a result of the repetitive behaviors). These symptoms may well arise from hyper-functioning of microcircuits (see section Prenatal Valproic Acid Exposure in Rats), and hypo-functioning of macrocircuits (as reflected by decreased white matter and connectivity in brains of ASD patients (Kana et al., 2011). The hyper-function of microcircuits may contribute to hyper-perception, hyper-attention, hyper-memory and hyper-emotionality. These symptoms may on their turn contribute to the progression of the disease, as overly strong reactions to experiences may become more and more extreme with each new experience especially when these experiences are emotionally charged. This may lead to obsessively detailed information processing. Due to hypo-functioning of macrocircuits this information is fragmented, leading to an inability to place the information in a broader context. Hence, the autistic patient is trapped into a limited but highly secure internal world with minimal extremes and surprises (Markram and Markram, 2010), as expressed by the DSM IV ASD core symptoms.

## The development of the serotonergic system

### The placenta as exogenous source of serotonin

Serotonergic neurons appear early during brain development, already releasing 5-HT before the establishment of conventional synapses as most of the axonal network maturation is achieved after birth in rodents. The function of this 5-HT release is to amplify its own synthesis and increase axon outgrowth (De Vitry et al., 1986; Witteveen et al., submitted). However, the influence of 5-HT is effective even before its neurons are born in the raphe nucleus. This suggests the need of an exogenous source of 5-HT at least during the early developmental stages. Synthesis of 5-HT requires two tryptophan hydroxylase (TPH) enzymes; TPH1 which is located in the pineal gland and gut enterochromaffin cells, and TPH2 which is restricted to the raphe nuclei and enteric nervous system. During development, expression of the transcripts starts at embryonic day (E) **10.5** for TPH2 and at **E12.5** for TPH1 (Cote et al., 2007). Before this stage, serotonergic signaling molecules, like the 5-HT2B receptor and plasmalemmal 5-HTT (E8–9) are already present (Buznikov et al., 2001). So the influence of 5-HT precedes that of its production. Since sites of earlier serotonin synthesis have not been found, the main source during that period has been shown to be maternal as the placenta is a source of serotonin (Cote et al., 2007; Bonnin et al., 2011; see Velasquez et al., 2013). Indeed, the essential amino acid tryptophan, which is the precursor of 5-HT, is present in placental tissue during E10.5–E14.5, which gives the placenta the necessary machinery to synthesize 5-HT. The capacity for placental 5-HT synthesis peaks at E14.5, suggesting that the placental source of 5-HT is of most importance in the period of early development (Bonnin et al., 2011), especially the forebrain. The mid/hindbrain, on the other hand, solely receives 5-HT input from the serotonergic neurons that arise at E10.5 in the dorsal raphe nuclei (for review and figures see van't Hooft and Smidt, submitted), which suggests a smaller importance of placental 5-HT for the development of the mid/hindbrain (Bonnin and Levitt, 2011). Hence, alterations in placental 5-HT likely will affect the early development of the forebrain, whereas genetic alterations in serotonergic genes are expected to affect the mid/hindbrain, as well as the forebrain in later developmental stages.

### The serotonin transporter (5-HTT)

A central position in the functioning of the serotonergic system is the 5-HTT. The 5-HTT is located in the plasma membrane of presynaptic nerve terminals from which 5-HT is released. It clears 5-HT from the extracellular space by reuptake mechanisms and thereby regulates serotonergic neurotransmission (Haenisch and Bonisch, 2011). There is only one gene encoding the 5-HTT, which is found in the central nervous system as well as peripheral tissue (Homberg et al., 2010). Expression of the 5-HTT gene starts in the serotonergic neurons of the mouse dorsal raphe nucleus at E11. By E16–E20 5-HTT is expressed in a multitude of brain regions including non-serotonergic ones such as the ganglionic eminence, thalamus, olfactory bulb, and cortex (Zhou et al., 2000). Around the second postnatal week in rodents, when neural circuits are pruned, 5-HTT expression declines in these non-serotonergic areas (Homberg et al., 2010), while it is maintained in the dorsal raphe nucleus throughout lifetime. The transient expression in various brain areas exclusively during their critical phase of development suggests that the 5-HTT plays an essential role in the establishment of brain circuits.

## 5-HTT gene variance in humans and rodents

### The human 5-HTT polymorphism

Abnormalities in 5-HTT function are implicated in ASD (BOX 1) by studies reporting reduced 5-HTT density in the frontal cortex of ASD patients (Makkonen et al., 2008; Nakamura et al., 2010) but see (Azmitia et al., 2011). The risk of abnormal functioning of 5-HTT can be increased by the genetic background of an individual. The most commonly studied genetic aberration of the 5-HTT is the 44-base pair insertion/deletion polymorphism in the promoter region of the gene (5-HTTLPR) (Champoux et al., 2002). The short (s) allelic variant of 5-HTTLPR is associated with a decrease in 5-HTT transcription (Heils et al., 1997), which presumably leads to decreased expression and function of 5-HTT. Since the 5-HTT is responsible for the reuptake of 5-HT, reduced availability of 5-HTT may lead to increased extracellular 5-HT levels. This, however, cannot be directly investigated in the human brain with the currently available methodologies (Holmes et al., 2003a; Homberg and Lesch, 2011). Regardless of whether or not the 5-HTTLPR s-allele is associated with increased 5-HT levels in the brain, the 5-HTTLPR s-allele has been associated with anxiety-related traits like neuroticism (Munafo et al., 2009). A recent meta-analysis revealed that the 5-HTTLPR s-allele is associated with a bias toward negative environmental stimuli (Kwang et al., 2010; Thomason et al., 2010; Fox et al., 2011; Pergamin-Hight et al., 2012), which may explain the association between the s-allele and anxiety-related traits. There are several indications that the 5-HTTLPR s-allele is indeed a strong genetic risk factor for ASD. For instance, the s/s genotype was found to be highly significantly associated with ASD (Devlin et al., 2005), albeit this association was dependent on the nature of ethnic populations (Huang and Santangelo, 2008; Arieff et al., 2010). It has also been demonstrated that the 5-HTTLPR s-allele is specifically associated with the failure to use non-verbal communication to regulate social interactions in ASD patients (Brune et al., 2006). Furthermore, the increased platelet serotonin level as has been consistently found in a fraction of autistic patients is linked to 5-HTTLPR genotype (Coutinho et al., 2004) but see (Betancur et al., 2002). Yet, recently it was reported that according to mothers' ratings children with the 5-HTTLPR l/l genotype had more severe ASD social deficits than 5-HTTLPR s-allele carriers (Gadow et al., 2013). It is possible that factors like ethnic background, scoring method, read-outs and age, contribute to such inconsistent findings.

Presumably, the presence of the 5-HTTLPR s-allele affects the structure and function of the early brain in such a way that it is more sensitive to adverse environmental stimuli like stress and/or that connectivity between brain regions is altered. It has been well-established that the 5-HTTLPR s-allele is associated with heightened reactivity of the amygdala in response to emotional stimuli (Hariri et al., 2002; Thomason et al., 2010). The amygdala plays a central role in emotional vigilance, particularly toward stimuli with a negative valence. Yet, the amygdala is also essential in social interactions and indeed, it plays a critical role in orienting gaze and attention to socially salient stimuli (Birmingham et al., 2011). Furthermore, ASD patients show increased amygdala activity during face processing (Monk et al., 2010; Kliemann et al., 2012). Hence, amygdala hyper-reactivity in association with the 5-HTTLPR s-allele may relate to both heightened emotional processing and social impairments, although it remains to be investigated whether these processes are based on similar mechanisms. Another neuronal phenotype associated with the 5-HTTLPR s-allele is a structural (Pacheco et al., 2009) and functional (Pezawas et al., 2005) uncoupling between the prefrontal cortex (PFC) and amygdala. Given that the PFC exerts an inhibitory control over the amygdala, a reduction in this inhibitory control is hypothesized to contribute to impaired emotion regulation in 5-HTTLPR s-allele carriers (Pezawas et al., 2005; Hariri and Holmes, 2006; Canli and Lesch, 2007; Homberg and Lesch, 2011), and thereby anxiety-related traits. Interestingly, independent from the 5-HTTLPR s-allele genotype, studies also revealed an association between altered prefrontal-amygdala connectivity and ASD. More specifically, in the so-called salience network there was a reduced connectivity between the insula and amygdala, which are considered as social brain regions (von dem Hagen et al., 2012). Furthermore, a social network involving the middle temporal gyrus, fusiform gyrus, amygdala, mPFC, and inferior frontal gyrus displayed reduced effective connectivity in ASD patients when exposed to facial expression (Sato et al., 2012). The 5-HTTLPR s-allele is also associated with increased cerebral cortical gray matter volumes in young male children with ASD (Wassink et al., 2007), for which the functional implications are unfortunately unclear. Finally, a core structural phenotype associated with ASD is decreased cortico-cortical connectivity, due to corpus callosum abnormalities. Indeed, ASD has consistently been linked with significantly less white matter density in the (anterior part of the) corpus callosum (Frazier and Hardan, 2009; Shukla et al., 2010; Hong et al., 2011; Schipul et al., 2012), suggesting aberrant long-range corticocortical connectivity. As to whether also the 5-HTTLPR s-allele is directly associated with corpus callosum changes remains to be determined. Given that there is active 5-HT uptake in the corpus callosum (Reyes-Haro et al., 2003), it is well-conceivable that the 5-HTTLPR affects corpus callosum connectivity.

### 5-HTT knockout mice and rats

Human studies have significantly advanced our understanding of the neural and behavioral phenotypes associated with the 5-HTTLPR, and thereby the possible role of 5-HT in neurodevelopment. However, detailed understanding of the neural correlates of the behavioral manifestations is limited due to inaccessibility of the human brain. As mentioned before, the 5-HTTLPR s-allele can be mimicked by a targeted reduction of the serotonin transporter gene in rodents (5-HTT^−/−^) (Holmes et al., 2003a). These animals exhibit high extracellular 5-HT levels due to impaired 5-HT clearance in the presynaptic nerve terminal (Mathews et al., 2004), and due to reduced 5-HT reuptake and limited 5-HT recycling in the presynaptic nerve terminal, serotonin synthesis is increased (Kim et al., 2005; Haenisch and Bonisch, 2011). Dorsal raphe neurons in 5-HTT^−/−^ mice show a reduced firing rate as well as desensitization and down-regulation of somatodendritic 5-HT_1A_ receptors, which exert an inhibitory control over raphe action potential firing activity (Lira et al., 2003). Postsynaptic 5-HT_1A_ receptors expressed in target regions of the dorsal raphe neurons, such as the frontal cortex, amygdala, septum, and hypothalamus, are decreased as well (Holmes et al., 2003a,b). These changes are likely compensatory adaptations in response to high extracellular 5-HT levels. Finally, there is convincing evidence that BDNF (brain-derived-neurotrophic factor) mRNA and protein levels are decreased in the PFC and hippocampus of 5-HTT^−/−^ rats (Molteni et al., 2010), which may correspond to the lower serum BDNF levels as observed in children with ASD (Correia et al., 2010; Al-Ayadhi, 2012). Given the role of BDNF in neuroplasticity, the lower availability of BDNF may contribute to the structural and functional changes in corticolimbic structures and white matter tracks in 5-HTTLPR s-allele carriers (section The Human 5-HTT Polymorphism) and 5-HTT^−/−^rodents (this section).

At the behavioral level, 5-HTT^−/−^ rodents show striking similarities with phenotypes observed in 5-HTTLPR s-allele carriers. For instance, 5-HTT^−/−^ mice show a reduction in exploratory locomotion in a light/dark exploration and in the homecage emergence test, as well as reduced open arm exploration in the elevated plus maze test (Haenisch and Bonisch, 2011). Since the reduction in activity is not due to impaired motor function, these results suggest an increase in anxiety-like behavior in 5-HTT^−/−^ mice (Holmes et al., 2003b). Also 5-HTT^−/−^ rats show anxiety-related symptoms in these behavioral tests, but without hypoactivity (Olivier et al., 2008). Whereas these behavioral tests are species specific, the finding that 5-HTT^−/−^ mice and rats, as well as human 5-HTTLPR s-allele carriers show impaired fear extinction (recall) (Garpenstrand et al., 2001; Wellman et al., 2007; Narayanan et al., 2011; Nonkes et al., 2012) implies that the role of 5-HTT in emotional control is highly conserved across species. Also striking is the finding that 5-HTT^−/−^ rodents consistently show a reduction in social interactions, which fit well with the pro-social behaviors reported for 5-HTTLPR s-allele carriers (Kiser et al., 2012). Furthermore, in line with the repetitive behaviors displayed by ASD patients (Pierce and Courchesne, 2001), 5-HTT knockout mice displayed higher frequencies of self-grooming than their wild-type littermates (Kalueff et al., 2010; Lewejohann et al., 2010). In the domain of communication, which is affected in ASD, it has been reported that wild-type mice show more ultrasonic vocalizations (USVs) within the 20–40 kHz range than prenatally stressed animals of both 5-HTT^+/+^ and 5-HTT^+/−^ genotypes, as well as non-stressed 5-HTT^+/−^ animals (Jones et al., 2010). Furthermore, 5-HTT^−/−^ rats show reduced prepulse inhibition (Page et al., 2009), implying the sensorimotor integration is impaired in these animals, such that they are unable to efficiently select sensory information from the external world. 5-HTT^−/−^ mice also show a reduced performance in the gap test measuring the functioning of the whiskers (Pang et al., 2011). These mice reach a smaller gap distance in this task, suggesting that their vibrissa related tactile perception is less sensitive compared to those of control animals. Finally, 5-HTT knockout mice display reduced inflammatory (Palm et al., 2008) and thermal (Vogel et al., 2003) pain. Given that ASD is characterized by impairments in social interaction, perceptual changes, as well as anxiety, one may argue that 5-HTT^−/−^ rodents very well-model a variety of phenotypes relevant for ASD. Notably, the interpretation of the (endo)phenotypes of 5-HTT knockout rats in the context of ASD is mainly based on face validity (Homberg, 2013). Furthermore, there are many factors like gender, age, gene × environment that influence behavior and have not and currently cannot be taken into account because of limited available information (Kas et al., 2011). Nonetheless, because the similarities between 5-HTT knockout (endo)phenotypes and those of the VPA ASD rat model (see section Prenatal Valproic Acid Exposure in Humans) is striking (Markram et al., 2007).

**Figure 2:**
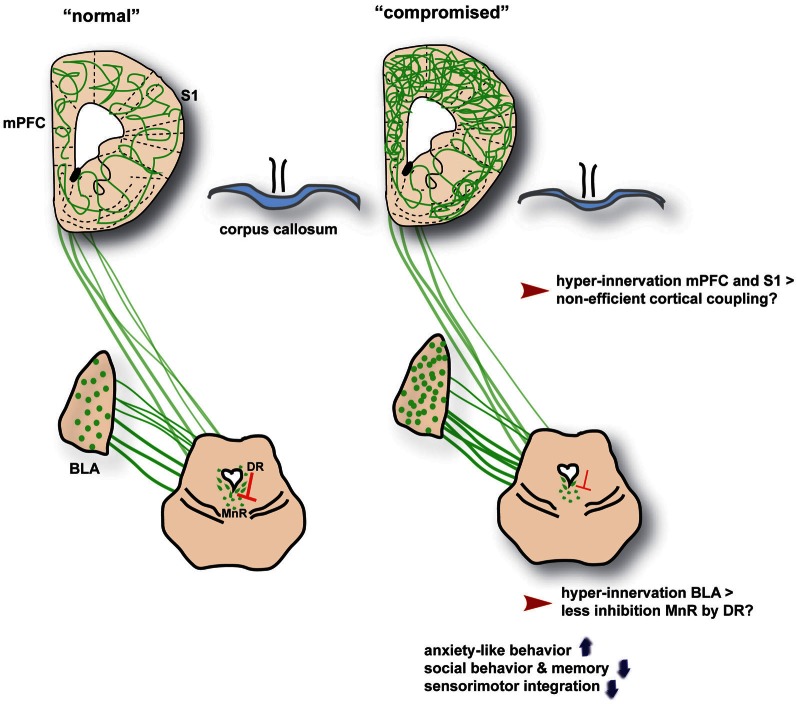
**A gross overview of the neural changes associated with increased neurodevelopmental serotonin levels across the 5-HTT, SSRI and VPA conditions (termed as “compromised”).** BLA, basolateral amygdala; DR, dorsal raphe nucleus; MnR, median raphe nucleus; S1, somatosensory cortex; mPFC, medial prefrontal cortex. The interaction between the DR and MnR is based on findings in 5-HTT knockout rats by Witteveen et al., submitted.

Although fMRI studies in rodents are hampered by the need for anesthetics in the MRI scanner, *ex vivo* immunostaining experiments have revealed morphological alterations in prefrontal regions and the amygdala of 5-HTT^−/−^ animals. For instance, excitatory pyramidal neurons in the amygdala and PFC of 5-HTT^−/−^ mice showed increased dendritic branching and an increased number of spines (Wellman et al., 2007). The early guidance and innervation of the mPFC pyramidal neurons by 5-HT projections from the raphe seem to be affected as well in 5-HTT^−/−^ rats as was shown by Witteveen et al. (submitted). It has also been reported that 5-HTT^−/−^ mice display increased cell density in the neocortex (Altamura et al., 2007), which may correspond to the increased gray matter found in s-allele ASD patients (Wassink et al., 2007). Furthermore, corpus callosum connectivity is reduced in 5-HTT^−/−^ rats, as measured by Diffusion Tensor Imaging (DTI) (Van der Marel et al., 2013) (Figure 2). This was noted at the level of the genu of the corpus callosum, which connects the prefrontal cortices, as has also been observed in ASD patients (Hardan et al., 2000; Vidal et al., 2006). Perhaps the most distinct morphological and functional alterations that have been reported in 5-HTT^−/−^ rodents involve the barrel cortex, which is part of the primary somatosensory cortex representing the whiskers. 5-HTT^−/−^ rats and mice show a distorted or nearly absent barrel pattern in cortical layer IV (Persico et al., 2000) (Miceli et al., submitted). Furthermore, Esaki et al. (2004) demonstrated that glucose uptake in the barrel cortex is significantly reduced in these mice, implying that the barrel cortex is also functionally impaired (Esaki et al., 2004). These changes may be related to altered (netrin-1-dependent) guidance of thalamocortical afferents (TCAs), which project to the barrels [see section The Serotonin Transporter (5-HTT)]. These TCAs appear less mature and less topologically organized in 5-HTT^−/−^ mice and rats (Cases et al., 1998). Given that ASD (Marco et al., 2012) and potentially depression (Kundermann et al., 2009) are associated with blunted (somato)sensory responses (section Perinatal SSRI Exposure in Humans) these 5-HTT^−/−^ findings are of great value to increase our understanding of the pathophysiology of these psychiatric conditions.

## Antidepressant (SSRI) exposure

### Perinatal SSRI exposure in humans

SSRIs are the most frequently prescribed antidepressants to help overcome depression and anxiety-related disorders. Their main target is the 5-HTT, which is inhibited by SSRI, leading to a pharmacologically induced increase in 5-HT levels in the extracellular space. During pregnancy women have an increased risk to develop depression-like disorders, with reports of depressed pregnant women ranging between 9 and 16% (Nonacs et al., 2005; Ververs et al., 2006; Field, 2010; Gentile and Galbally, 2011). Given that depression is associated with an increased risk of preterm delivery, low birth weight, operative delivery, and admission of the newborn to the neonatal intensive care unit (Chung, 2001; Bonari et al., 2004; Field, 2010), antidepressant treatment is mandatory. With only few side effects reported in adults, and therefore regarded safe, SSRIs are the drug of choice for the treatment of depression during pregnancy. As such, around 25% of the depressed women continue SSRI use, and another 0.5% start using them during pregnancy (Ververs et al., 2006). However, SSRIs cross the placenta (Rampono et al., 2004) with SSRI transfers ranging between a ratio of 52 and 72% (Rampono et al., 2004). After birth, exposure of the offspring to SSRIs also occurs through breast milk during the neonatal period of breast feeding (Homberg et al., 2010; Capello et al., 2011). This is problematic, because SSRI-induced rises in 5-HT levels can affect neurodevelopmental programming. Clinical data have already emphasized the potential hazards of prenatal SSRI exposure [for review see (Alwan and Friedman, 2009; Gentile and Galbally, 2011)]. The symptoms that have been noted in SSRI exposed newborns of depressed mothers compared to non-exposed newborns of depressed mothers include tremor, hypoglycemia, sudden infant death, pulmonary hypertension, rigidity, low Apgar scores, startles, tremors, back-arching, and hypertonic reflexes (Laine et al., 2003; Moses-Kolko et al., 2005; Chambers et al., 2006; Salisbury et al., 2011; Colvin et al., 2012), lower birth weights and preterm births (Lee, 2009; Oberlander et al., 2009; Grzeskowiak et al., 2012), poor feeding, weaker or absent cry, tachypnea and an increase in motor activity (Zeskind and Stephens, 2004). Additionally, it has been reported that infants show blunted somatosensory responses upon prenatal SSRI exposure(Oberlander et al., 2005), poorer psychomotor development (Casper et al., 2003, 2011), an increased risk for ASD symptoms (Croen et al., 2011), and early death (Colvin et al., 2012). Furthermore, children exposed *in utero* to SSRIs that developed a neonatal abstinence syndrome were at an increased risk for social-behavioral abnormalities (Klinger et al., 2011). Given that 5-HTT^−/−^ rodents display an impaired whisker dependent tactile perception and reduced social interactions (a core symptom of ASD) (see Box 1), there appears to be a striking resemblance between the neurodevelopmental consequences of prenatal SSRI exposure and genetic 5-HTT down-regulation. This is further supported by rodent perinatal SSRI exposure studies, as discussed in detail below.

### Perinatal SSRI exposure in rodents

Whereas human studies are hampered by ethical and time-related limitations, rodents are well-suited to obtain insight in the long-term consequences of prenatal SSRI exposure. Notably, it has been reported that the placental transfer of fluoxetine is 70–80% in rodents, and thereby comparable to values reported in humans (Noorlander et al., 2008; Olivier et al., 2011). This has been shown to have profound consequences for the structural and functional organization of the developing brain of the fetus. In rats prenatal SSRI exposure does not only block 5-HTT activity but also reduces its expression. Furthermore, 5-HT_2A_ and 5-HT_2C_ receptor density is reduced, indicating an overall decrease of 5-HT activity. In addition, the expression of Tph2, which is crucial for the synthesis of 5-HT, is reduced in the raphe nuclei after neonatal SSRI treatment (Maciag et al., 2006). Also the number of 5-HTT and TpH2 density is reduced in the raphe nuclei of perinatally SSRI exposed rats (Simpson et al., 2011). Autistic children show decreases of α-methyl-l-tryptophan, which is an analogue to the 5-HT precursor tryptophan, in the left cortical hemisphere and exhibit a higher prevalence of severe language impairment, whereas those with decreases in the right cortical hemisphere more frequently display left and mixed handedness (Chandana et al., 2005). Additionally, depletion of tryptophan, the precursor of 5-HT, has been found to increase various stereotyped behaviors in autistic children (McDougle et al., 1996). Yet, early-life fluoxetine exposure resulted in the long-term up-regulation of BDNF expression in adult mice, which seemingly contrast the BDNF down-regulation in 5-HTT^−/−^ rats (section 5-HTT Knockout Mice and Rats) and ASD patients (Correia et al., 2010; Al-Ayadhi, 2012).

Also comparable to the human situation is the finding that rats treated with fluoxetine during pregnancy delivered smaller pups (Vorhees et al., 1994). It is well-known that weight loss is a side-effect of fluoxetine in non-pregnant women and in men; therefore these results could be related to lowered maternal weight gain, which in turn could limit fetal growth. Furthermore, Noorlander and colleagues (2008) found that the majority of the mouse pups that were exposed to fluoxetine during pregnancy died postnatally of severe heart failure caused by dilated cardiomyopathy. Similar effects were found in rats that were exposed to paroxetine treatment during the last week of gestation, which led to a shortened gestational length, reduced birth weight and a 10-fold rise in neonatal mortality (Van den Hove et al., 2008). These findings may match the increased risk for mortality in prenatally SSRI exposed children (Colvin et al., 2012). Taking advantage of the relative short life time of rodents, it has been demonstrated that prenatal or early postnatal (P4-P21) SSRI treatment leads to anxiety- (Ansorge et al., 2004; Smit-Rigter et al., 2012) and depression-related (Hansen et al., 1997; Popa et al., 2008) phenotypes during adulthood. Furthermore, in correspondence with the repetitive behavior phenotype of ASD, prenatal SSRI exposure has been reported to increase grooming and digging behavior (Rodriguez Echandia et al., 1988), as was reported in 5-HTT^−/−^ mice (section 5-HTT Knockout Mice and Rats). Besides, evidence is now accumulating showing that prenatal SSRI treatment leads to blunted somatosensory responses and decreases in social behavior, as reported for 5-HTT^−/−^ rats. Regarding the somatosensory responses, (Lee, 2009) showed that postnatal SSRI (fluoxetine) treatment from P0-P7 decreased performance in the whisker-dependent gap test. This effect could be mimicked by clipping the wishers, and matches the decreased gap test performance reported in 5-HTT^−/−^ mice (Pang et al., 2011). Also prepulse inhibition was reduced in prenatally fluoxetine exposed rats (Olivier et al., 2011), indicative for an impairment in sensorimotor integration. Finally, prenatal (E11-delivery) and perinatal (P4/8-P21) SSRI treatment have been demonstrated to decrease social play behavior in juvenile rats (Olivier et al., 2011; Simpson et al., 2011), aggressive behavior (Manhaes de Castro et al., 2001), and sexual behavior (Maciag et al., 2006). Again, the reduction in social behavior across ages is consistent with findings in 5-HTT^−/−^ rodents, as well as the effects of prenatal SSRI exposure in humans.

These robust behavioral alterations due to developmental SSRI exposure must be reflected by changes in the wiring of the brain. To provide a link with the blunted functioning of the whisker related somatosensory system, the structure and physiological properties of the barrel cortex and its afferent thalamocortical connections have been in the focus of several studies. Fluoxetine, when applied to rats during PND 0–6, leads to a reduction in the complexity of TCA projections into the barrel cortex. On the intracortical target side, excitatory spiny stellate cells within the layer IV barrel structures possess a reduced dendritic span and arborization (Lee, 2009). Comparable findings were obtained by Xu and coworkers (2004), who found that exposure to the SSRI paroxetine in rats from PND 0 till PND 8 affected the refinement, but not the formation, of dense clusters of the TCA's in the layer IV of the barrel cortex (Xu et al., 2004). Thus, developmental increases in 5-HT levels lead to substantial alterations in the somatosensory system and most likely explain the blunted tactile perception as reported in postnatal SSRI treated rats (Lee, 2009). Of further interest, it has been demonstrated that postnatal SSRI treatment in rat pups altered the myelination of axons in the corpus callosum and interfered with oligodendrocyte (OL) soma morphology. OLs showed hypo- and hypermyelination (Simpson et al., 2011), and the processes of OL progenitor cells were shortened, distorted, and/or polarized. Because the corpus callosum connects hemispheres, it was also investigated whether the aberrant morphology of OLs affected cortico-cortical connectivity. Retrograde tracer studies revealed a reduction in the connectivity between the primary somatosensory cortices across the hemispheres. This was more pronounced for layers II/III than for layer IV (barrel cortex) (Simpson et al., 2011). Although this connectivity has not been investigated in human 5-HTTLPR-s allele carriers, 5-HTT^−/−^ rats, and ASD patients, the decrease in corpus callosum connectivity found in 5-HTT^−/−^ rats and ASD patients (see section 5-HTT Knockout Mice and Rats) may suggest that developmental increases in 5-HT levels affects myelination, and thereby long-distance connectivity in the brain. Moreover, the structural uncoupling between the PFC and amygdala in 5-HTTLPR s-allele carriers (Pacheco et al., 2009; section 5-HTT Gene Variance in Humans and Rodents) implies that besides the corpus callosum other white matter tracks are altered by high developmental 5-HT levels, too. Because complex behaviors like social behavior requires the correct integration of information derived from several brain regions, it is conceivable that alterations in myelination and thereby the long ranging connectivity between hemispheres and brain regions contribute to the behavioral deficits seen in ASD.

## Prenatal valproic acid exposure

### Prenatal valproic acid exposure in humans

Besides prenatal SSRI exposure, prenatal exposure to VPA leads to ASD-related symptoms in humans and rats. VPA is a mood stabilizing drug primarily used in the treatment of bipolar disorder and epilepsy (Markram et al., 2007). It inhibits the enzyme histone deacetylase, which mediates epigenetic processes through acetylation of histone proteins. A decrease in histone acetylation, as may be induced by VPA treatment, makes the DNA less accessible to the transcriptional machinery and is hypothesized to be associated with a decrease in gene expression (Yildirim et al., 2003). Depending on the timing of decreased histone acetylation, this leads to a cascade of neuropathologies including ASD. Indeed, case studies have shown that prenatal VPA exposure is likely to induce ASD (Christianson et al., 1994; Williams and Hersh, 1997; Williams et al., 2001). An increased incidence of ASD is specifically found following fetal exposure to the agent around the time of neural tube closure. It is worth mentioning that this finding has led to the hypothesis that ASD may be caused by brainstem injury during embryonic development (Rodier et al., 1996; Arndt et al., 2005). Given that the raphe nuclei are located in the brainstem and start to develop at the time of neural tube closure, the serotonergic system is one possible target of VPA-mediated alterations in gene expression.

### Prenatal valproic acid exposure in rats

Based on the human case studies, the VPA rat model for ASD has been established. In rats, the neural tube closes at E9. A single dose of VPA (350 mg/kg) administered to pregnant dams on E12.5 results in a decrease in social interactions, increase in repetitive behavior, enhanced anxiety, impaired fear extinction, and impaired pre-pulse inhibition (Schneider and Przewlocki, 2005; Schneider et al., 2006, 2007; Markram and Markram, 2010). Remarkably, these behavioral manifestations resemble those found in 5-HTT knockout (section 5-HTT Knockout Mice and Rats) and prenatally SSRI exposed (section Perinatal SSRI Exposure in Rodents) rodents. It has also been reported that prenatally VPA exposed rats failed to emit the characteristic 70 kHz USV preceding mating, and that pups show a reduction in distress calls (Gandal et al., 2010). Possibly this matches the finding that 5-HTT^+/−^ mice show decreased ultrasonic vocalization (section 5-HTT Knockout Mice and Rats). However, results are diverse since Felix-Ortiz and coworkers observed an increase of three specific forms of USVs on PND5 of VPA treated mice (Felix-Ortiz and Febo, 2012). Furthermore, VPA exposed rats show reduced pain sensitivity (Schneider et al., 2001; Schneider and Przewlocki, 2005), which may match the reduced pain perception observed in 5-HTT knockout mice (section 5-HTT Knockout Mice and Rats) as well as the blunted somatosensory responses as reported in prenatally SSRI exposed infants (section Perinatal SSRI Exposure in Humans) and rats (section Perinatal SSRI Exposure in Rodents) and 5-HTT knockout rats (section 5-HTT Knockout Mice and Rats). Recent studies provided direct evidence for VPA interfering with the serotonergic system. Kuwagata et al. (2009) showed that a VPA challenge at E11 was associated with abnormal migration of serotonergic neurons at the level of the pons, which coincidences with the appearance of serotonergic neurons at E10.5. Yet, others reported that the serotonergic system was pertubated after administration of VPA at E9, thus at an earlier developmental time point. It was found that VPA exposure at E9 was associated with an increase in 5-HT levels in the blood as well as the frontal cortex, hippocampus and cerebellum (Miyazaki et al., 2005; Dufour-Rainfray et al., 2010). VPA exposure in rats does not alter the number of serotonergic neurons, but their location is shifted more caudally within the dorsal raphe nucleus, probably caused by abnormal serotonergic neuronal differentiation and migration (Miyazaki et al., 2005; Tsujino et al., 2007). Finally, *in situ* hybridization experiments revealed lower cortical expression of BDNF mRNA in VPA exposed rats (Roullet et al., 2010), like 5-HTT^−/−^ rats (section 5-HTT Knockout Mice and Rats). In sum, the VPA rat model shares phenotypic similarities with the prenatal SSRI and 5-HTT knockout models, and possibly these models even share 5-HT-mediated structural changes (Figure 2).

From a mechanistic point of view it is striking that both 5-HTT knockout rats and VPA exposed rats show hyper-reactivity in cortical layers II/III, as revealed by electrophysiological recordings using multi electrode arrays (Rinaldi et al., 2007; Miceli et al., submitted). This can be caused by increased synaptic efficiency, hyper-connectivity, lack of proper inhibitory control, or by alterations in neuron density and morphology. Hyper-reactivity was also found in layers II/III of VPA exposed rats, as reflected by enhanced long-term potentiation (reflecting altered synaptic plasticity). Besides the cortex, amygdala hyper-connectivity and hyper-reactivity have been noted in VPA exposed rats. That is, neurons in the lateral amygdala were found to be hyper-reactive when electrically stimulated using the multi electrode array technology. It was also found that this was due to a reduction in inhibition (Markram et al., 2007). Furthermore, like in the cortex, long-term potentiation was increased in the amygdala of VPA exposed rats, indicative for hyper-plasticity. It is well-possible that this explains the enhanced fear memories (as reflected by impaired fear extinction) in VPA exposed rats. Although 5-HTT knockout mice show increased dendritic complexity of pyramidal neurons in the amygdala (Wellman et al., 2007) and increased cell density in the neocortex (Altamura et al., 2007), whether there are similarities at either of these levels in the 5-HTT knockout and VPA exposed rats remains to be established. *Vice versa*, the corpus callosum abnormalities reported in perinatally SSRI exposed rats (Simpson et al., 2011); section Perinatal SSRI Exposure in Rodents) and reduced corpus callosum connectivity found in 5-HTT rats (Van der Marel et al., 2013; section 5-HTT Knockout Mice and Rats) remain to be investigated in the VPA rat model.

As proposed by Markram et al. (2007), autism may be associated with excessive neuronal information processing and storage in (cortical) microcircuits. This may lead to hyper-perception, hyper-attention, and hyper-memory. Simultaneously, autism is associated with reduced long-distance cortical and subcortical connections, impairing the integration of different pieces of information, and thereby complex cognitive and social functions (Kana et al., 2011). In other words, autistic patients may experience the world intensely but fragmented. The impairments in social behavior may also arise from this fragmented intense world syndrome, as ASD patients may experience social cues overly intense while being unable to integrate these social cues as is needed for a proper understanding. This may lead to avoidance of eye and social contact. Of interest, we have suggested these behavioral manifestations also for 5-HTTLPR s-allele carriers, by using the term “hypervigilance” (Homberg and Lesch, 2011). We used this term—based on amygdala and prefrontal hyper-reactivity in fMRI studies—to explain why these individuals are supersensitive to adverse as well as rewarding environmental influences. 5-HTT knockout rats show stimulus-bound habitual-like behavioral responses (e.g., impaired goal-directed behavior in the reward devaluation task) (Nonkes et al., 2010, 2011, 2012), which also may be the consequence of a fragmented world: If the world is perceived as fragmented, it may be very effective to use conditioned cues as a hand-tight to behaviorally perform in a world consisting of a “chaos” of intense stimuli. The consequence, however, may be that this leads to behavioral persistence or repetitive behavior. In both 5-HTT knockout rodents and VPA exposed rats this for instance may be reflected by fear extinction failures. Also the lack of goal-directed behavior in 5-HTT knockout rats (Nonkes et al., 2010) implies that these animals are unable to update a previously acquired conditioned response. Possibly, this matches impairments in goal-directed behavior observed in ASD patients (Poljac and Bekkering, 2012). It would be intriguing to assess whether VPA exposed rats show similar phenotypes. There is, however, one paradox: Whereas the hypervigilance in 5-HTTLPR s-allele carriers (Jedema et al., 2010) and 5-HTT knockout rodents (Brigman et al., 2010; Nonkes et al., 2011) conveys increases behavioral flexibility, no changes in behavioral flexibility has been noted in prenatally SSRI treated animals (Ishiwata et al., 2005). Furthermore, VPA treatment during gestation caused a reduction in behavioral flexibility, (Stanton et al., 2007). Possibly, non-serotoninergic systems may be involved in the behavioral inflexibility observed in VPA exposed rats.

## Discussion

As we reviewed in this article, there is strong evidence that 5-HT plays a major role in the etiology of mood disorders and particularly ASD. Exposure to elevated levels of 5-HT over a long time period during crucial periods of brain development, or a fetal SSRI/VPA challenge during a critical developmental stage, causes alterations in the wiring of the brain, both at the microcircuit and macrocircuit level. This leads to rather consistent behavioral manifestations (Table 1), including decreased social interactions, increased anxiety-like behavior, and blunted (somato) sensory perception. The brain areas that mediate, at least in part, these behavioral manifestations include the (prefrontal/somatosensory) cortex and the amygdala. More specifically, it is likely that amygdala hyper-reactivity contributes to anxiety, enhanced fear memories, and social impairments in 5-HTTLPR s-allele carriers, 5-HTT knockout rodents, prenatally SSRI exposed animals and VPA exposed rats. Indeed, the amygdala is strongly implicated in signaling the emotional value or salience of environmental stimuli, including social stimuli (Adolphs, 2010). Furthermore, ASD is characterized by amygdala hyper-reactivity in response to adverse stimuli (Kleinhans et al., 2010; Weng et al., 2011). Changes in the organization of the “labeled line” of the somatosensory system are responsible for the alterations in sensory performance of 5-HTT knockout rodents, prenatally SSRI exposed animals, and possibly also in VPA exposed rats. Given that social behavior is strongly dependent on how social stimuli are perceived, for instance through the whiskers in rodents, hyper-reactivity in the somatosensory cortex may contribute to the social impairments in these models as well. Yet, it appears counterintuitively that cortical hyper-reactivity would contribute to blunted somatosensory responses and reduced social interactions, unless it represents a compensatory mechanism for reduced or diffuse sensory input, as described by Miceli et al. (submitted) based on neuroanatomical findings in 5-HTT knockout rats. As speculated (section Prenatal Valproic Acid Exposure in Rats), if environmental stimuli are perceived overly intense, these responses are driven by avoidance or withdrawal, as a self-protective mechanism.

**Table 1:**
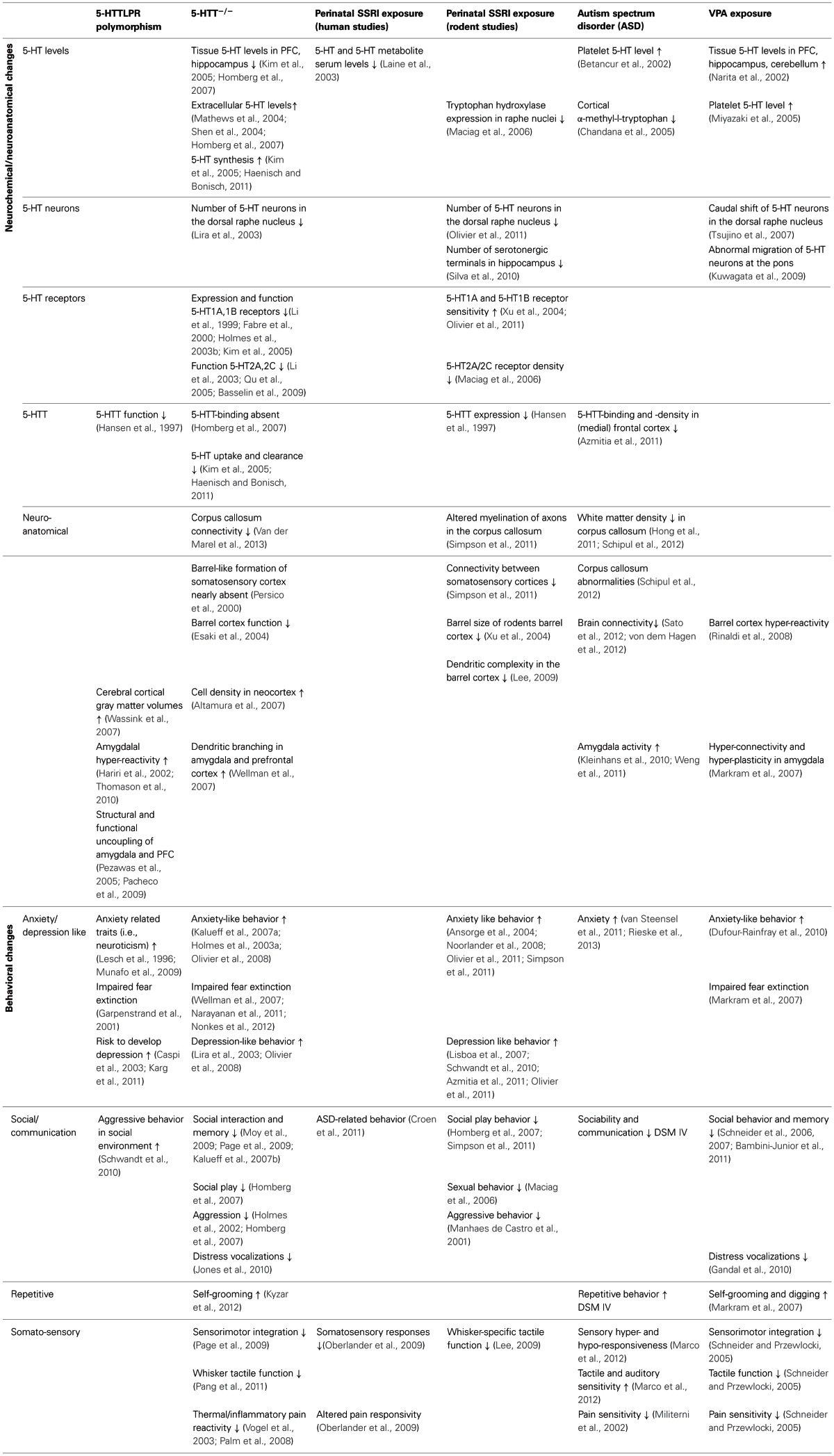
**Neuroanatomical and behavioral alterations in 5-HTTLPR s-allele carriers and 5-HTT−/− rodents, perinatally SSRI exposed humans and rats, ASD patients, and VPA exposed rats**.

To increase our understanding of the role of 5-HT in brain development, with important implications for ASD, it would be essential to fill in the “gaps” in Table 1, and to link structural phenotypes to behavior. To this end, new *in vivo* technologies like optogenetics will significantly increase the understanding of the physiological properties of specific cell-types in relation to behavior. E.g., if cortical layer II/III neurons show hyper-connectivity, optogenetically-mediated modulation in firing of specific excitatory or inhibitory neuron classes upon their activation in response to specific environmental stimuli (e.g., whisker stimulation) may help to remediate the somatosensory part of the intense world syndrome. Furthermore, using *in utero* electroporation, a technique that allows region specific gene manipulation in embryo's (Kolk et al., 2011), we might be able to understand through which 5-HT receptors 5-HT mediates its developmental effects. This information may eventually lead to pharmacological targets to steer the structural and behavioral consequences of high 5-HT levels during embryonic development.

In sum, comparing distinctive human conditions and animal models characterized by early life perturbations of the serotonergic system is a powerful approach to unravel the structural and behavioral consequences of high 5-HT levels during development. Indeed, similarities in brain and behavior in human subjects and animal models characterized by high 5-HT levels during development will strengthen the value of experimental findings and bring us closer to the answer how 5-HT during development contributes to ASD-related symptoms.

### Conflict of interest statement

The authors declare that the research was conducted in the absence of any commercial or financial relationships that could be construed as a potential conflict of interest.
